# Bifocal lesions have a poorer treatment outcome than a single lesion in adult patients with intracranial germinoma

**DOI:** 10.1371/journal.pone.0264641

**Published:** 2022-03-01

**Authors:** Yu-Mei Kang, Yi-Yen Lee, Shih-Chieh Lin, Feng-Chi Chang, Sanford P. C. Hsu, Chun-Fu Lin, Muh-Lii Liang, Hsin-Hung Chen, Tai-Tong Wong, Keng-Li Lan, Yee Chao, Yi-Wei Chen

**Affiliations:** 1 Division of Radiation Oncology, Department of Oncology, Taipei Veterans General Hospital, Taipei, Taiwan; 2 Faculty of Medicine, National Yang Ming Chiao Tung University, Taipei, Taiwan; 3 Institute of Clinical Medicine, School of Medicine, National Yang Ming Chiao Tung University, Taipei, Taiwan; 4 Division of Pediatric Neurosurgery, Neurological Institute, Taipei Veterans General Hospital, Taipei, Taiwan; 5 Department of Pathology and Laboratory Medicine, Taipei Veterans General Hospital, Taipei, Taiwan; 6 Department of Radiology, Taipei Veterans General Hospital, Taipei, Taiwan; 7 Department of Neurosurgery, Neurological Institute, Taipei Veterans General Hospital, Taipei, Taiwan; 8 Institute of Traditional Medicine, School of Medicine, National Yang Ming Chiao Tung University, Taipei, Taiwan; 9 Department of Oncology, Taipei Veterans General Hospital, Taipei, Taiwan; 10 Department of Medical Imaging and Radiological Technology, Yuanpei University of Medical Technology, Hsinchu City, Taiwan; Chang Gung Memorial Hospital and Chang Gung University, Taoyuan, Taiwan, TAIWAN

## Abstract

Intracranial germinoma (IG) rarely occurs in adults. Its optimal treatment strategy is unclear. We evaluated the outcomes of radiotherapy in adults with intracranial germinoma. Data of 29 adult patients (age, 18–52 years; median age, 24.3 years) with IG treated with radiotherapy at Taipei Veterans General Hospital were retrospectively reviewed. They were followed up for a median time of 5.9 years (range, 1.0–12.8 years). We used the Kaplan–Meier method to estimate the progression-free survival (PFS) and overall survival (OS), and univariate and multivariate Cox proportional hazards regression models to identify the factors affecting PFS. PFS and OS were compared between adult and pediatric patients with IG. The 1-, 3-, and 5-year PFS rates were 96.6%, 85.8%, and 77.8%, respectively, in the adult patients, and the OS rate were all 100%. Seven patients (24.1%) experienced recurrence, and in six of them, salvage therapy successfully controlled the disease. Two patients (6.9%) died after 5 years of follow-up due to disease progression and central pontine myelinolysis. In the univariate and multivariate Cox analysis, bifocal lesions had a significantly lower PFS than those with single lesions (*p =* 0.008). Kaplan–Meier survival analysis showed that adult patients had a poorer PFS (*p =* 0.06) and OS (*p =* 0.025) than pediatric patients. Our study showed bifocal lesions were associated with lower PFS than a single lesion among adult IG patients, and adult IG patients tended to have poorer PFS and OS compared to pediatric IG patients. For adult patients with bifocal IG, we recommend treatment with craniospinal irradiation, whole ventricle irradiation (WVI) with chemotherapy, or frequent spine images follow-up for patients who received only WVI.

## Introduction

Intracranial germ cell tumor (IGCT) is a rare type of cancer that affects approximately 0.6–1.0 per million people per year in the United States and Europe [1], and it represents 0.5–3% of primary neoplasms in North America and Europe [2]. Intracranial germinoma (IG) is a major type of IGCT that accounts for approximately 60–70% of IGCTs [3]. IGs are considered more common in East Asia [4]. However, recent literature has reported similar incidences in Japan and the United States [5]. It affects 0.5 per million people per year in the United States [5]. IGs generally occur during childhood and adolescence, and 90% of patients are diagnosed before the age of 20 years [6]. IGs in older adults are rare, with only a few case reports and one case series documented in literature [7–9]. Therefore, investigating the optimal treatment for adult patients with IGs is important.

Since IGs are highly sensitive to chemo- and radiotherapy (RT) [10], the treatment of non-metastatic IGs has currently evolved from surgery plus craniospinal irradiation (CSI) with high-dose focal RT to whole ventricle irradiation (WVI) with or without chemotherapy (CHT) [11]. Lo et al. proposed that gaps in scientific knowledge and lack of accordant management may potentially lead inferior survival for adolescents and young adults IG patients [12]. One study reported that older age contributes to inferior overall survival (OS) [13]. Another study showed that treatment modality, not the age, was an important predictor of inferior OS [14]. A study from Canada reported a large inconsistency in the management of an adolescent with pineal germinoma between a pediatric oncologist and an adult oncologist [12]. Therefore, establishing an optimum treatment strategy for adult IG is crucial.

Here, we present the treatment outcomes and predictive factors of 29 adults with IG treated with RT at our institute. To our knowledge, this is the largest study reporting treatment outcomes in adult patients with IG.

## Methods

### Clinical data collection

The records of adult and pediatric patients with IG treated with RT at the Taipei Veterans General Hospital between 2006 and 2016 were retrospectively reviewed. A total of 96 patients with IG were identified, and information regarding their age, sex, date of diagnosis, symptoms, neuroimaging data, laboratory results, treatment modality, failure patterns, recurrence date, last follow-up date, death date, and treatment-related toxicities was collected. Among all patients with IG, 29 adult patients (age ≥ 18 years) and 67 pediatric patients (age < 18 years) were identified. This study focused on analyzing the data of adults with IG.

The study endpoints were progression-free survival (PFS) and overall survival (OS). Tumor status was evaluated by experienced neuroradiologists based on the Response Evaluation Criteria in Solid Tumors guidelines. Local or regional recurrence or distant metastasis based on imaging or pathological findings was defined as a PFS event. Death related to the disease was defined as an OS event. Follow-up duration was defined as the period from the date of diagnosis to the date of either an OS or DSF event. Treatment-related toxicities were assessed using the Common Terminology Criteria for Adverse Effects version 5.0. Approval for this study was obtained from the Taipei Veterans General Hospital Institutional Review Board (IRB No.: 2020-06-009AC).

### Treatment strategy and therapeutic diagnosis

**Fig 1:**
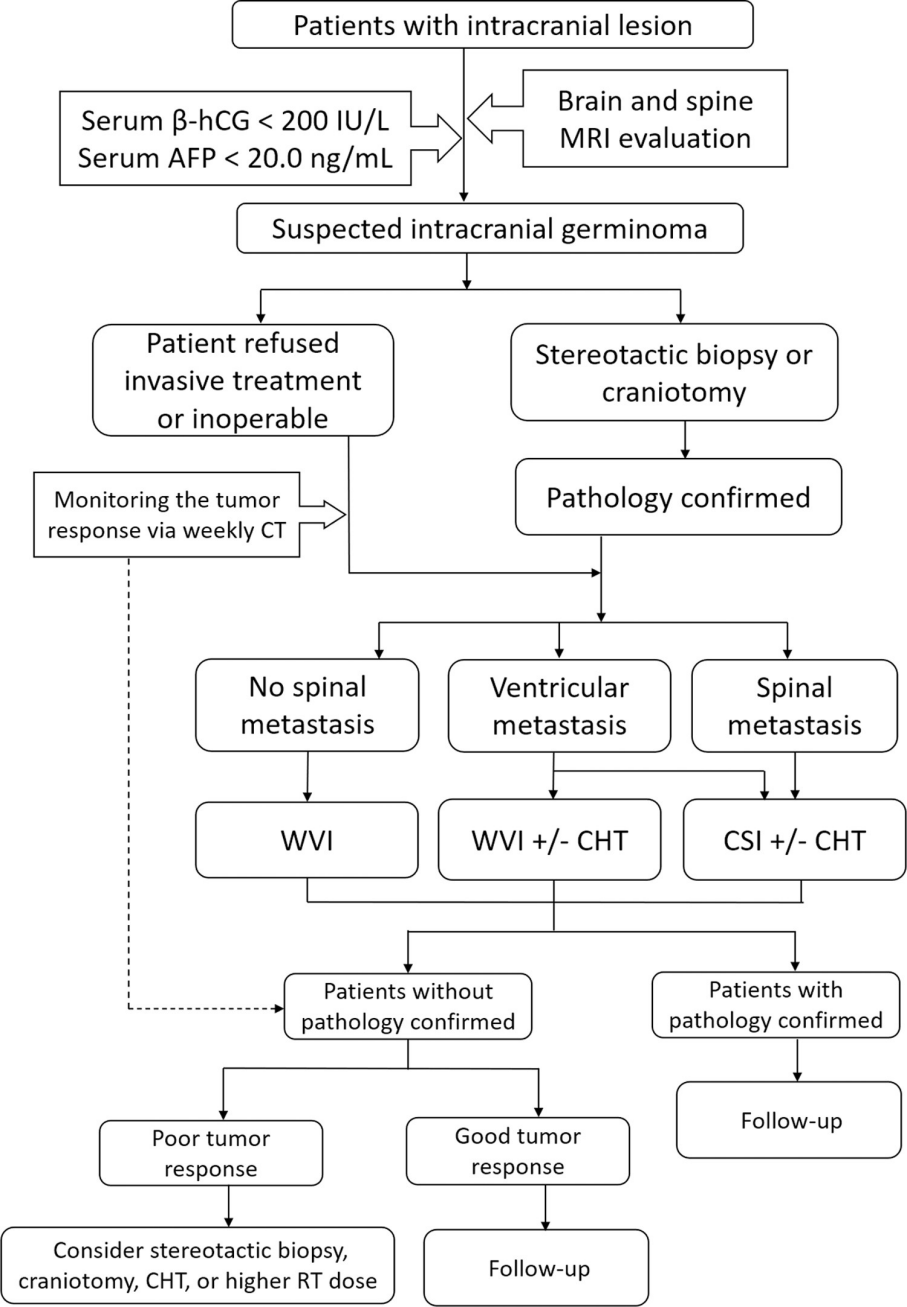
Treatment flow chart of intracranial germinoma patients.

The flow chart of the current treatment strategy used in our institute for patients with IG is described in Fig 1. Patients underwent neuro-imaging investigations and laboratory test for serum tumor marker, including serum alpha-fetoprotein (AFP) (<20.0 ng/mL) and beta-human chorionic gonadotropin (β-hCG) (<200 IU/L) to rule in IG. In our hospital, the treatment strategies for pediatric and adult patients with IG were identical. Stereotactic biopsy or craniotomy was more strongly suggested for adult patients suspected of having IG because this diagnosis is uncommon in the adult population. Samples from stereotactic biopsies or craniotomies were evaluated by an experienced neuropathologist.

For patients with IG confirmed through craniotomy or stereotactic biopsy, CSI was recommended in cases with spinal metastasis at diagnosis, while WVI or CSI could be considered for patients with ventricular metastasis but no spinal metastasis at diagnosis. WVI was recommended only for patients without spinal or ventricular metastasis. In WVI, the tumor and whole ventricle areas were delineated on brain magnetic resonance imaging (MRI) with a 1.0–1.5 cm margin as clinical target volume (CTV)-low, and received a radiation dose of around 24 Gy. A CTV-high was defined with a margin of 1.0 cm around the tumor, adding 6–12 Gy as primary boost (PB) after WVI. The daily RT fraction size was 1.8–2.0 Gy.

For patients in whom craniotomy or stereotactic biopsy was not performed due to clinical conditions or personal reasons, the tumor response after receiving RT was monitored weekly using computed tomography (CT). Significant tumor shrinkage was observed after RT initiation during the second or third week of treatment. The treatment strategy was the same as previously described for patients with pathological proof of IG. However, if the tumor response was poor, after discussions in multidisciplinary meetings, stereotactic biopsy, craniotomy, CHT, or a higher RT dose were recommended.

As for CHT, carboplatin and etoposide for six to ten cycles would be arranged if ventricular or spinal seeding was suspected at diagnosis and adjusted according to the patient’s clinical condition and personal willingness.

### Statistical analysis

The Kaplan–Meier estimate was used to analyze PFS and OS. Recurrences, including local, regional, or distant metastases, were considered events associated with PFS. Deaths related to IG or treatment were identified as events associated with OS. The differences between survival curves were analyzed using the Kaplan-Meier method and the log-rank test. Univariate and multivariate Cox regression analyses were used to identify the factors affecting PFS. All analyses were performed using the R project (version R-3.6.3; http://www.r-project.org). Statistical significance was defined as a two-sided *p*-value <0.05.

## Results

### Patient characteristics

The demographic information of the 29 adult and 67 pediatric patients with IG is shown in Table 1. For adult patients with IG, the median age at diagnosis was 24.3 years (range, 18.3–51.9 years). There were three women (10.3%) and 26 men (89.7%). At the time of diagnosis, three patients (10.3%) had spinal seeding and five patients (17.2%) had ventricular seeding. The tumor was located in the pineal region in eight patients (27.6%), suprasellar region in 11 patients (37.9%), both pineal and suprasellar regions (bifocal lesions) in eight patients (27.6%), and basal ganglia in two patients (6.9%). Tumor marker examination revealed normal levels of serum AFP (<20.0 ng/mL) in all patients. The serum levels of β-hCG were relatively high (10–200 IU/L) in two patients (6.8%) and low (<10 IU/L) in 27 patients (93.1%).

**Table 1:** Demographic characteristics of adult intracranial germinoma patients.

Characteristic	Adult IG patients (N = 29)	Pediatric IG patients (N = 67)
**Median age (years) (Range)**	24.3 (18.3–51.9)	12.6 (4.0–17.0)
**Median follow-up (years) (Range)**	5.9 (1.0–12.8)	7.4 (1.0–12.7)
**Sex**		
**Male**	26 (89.7%)	50 (74.6%)
**Female**	3 (10.3%)	17 (25.4%)
**Initial disease status**		
**Localized disease**	21 (72.4%)	55 (82.0%)
**Ventricular seeding**	5 (17.2%)	10 (14.9%)
**Spinal seeding**	3 (10.3%)	2 (3.0%)
**Tumor area**		
**Pineal area**	8 (27.6%)	22 (32.8%)
**Suprasellar**	11 (37.9%)	24 (35.8%)
**Bifocal**	8 (27.6%)	4 (6.0%)
**Basal ganglia**	2 (6.9%)	17 (25.4%)
**RT field**		
**Focal (Tumor only)**	2 (6.9%)	8 (11.9%)
**WVI+ PB**	20 (69.0%)	53 (79.1%)
**WB+ PB**	1 (3.4%)	3 (4.5%)
**CSI+ PB**	6 (20.7%)	3 (4.5%)
**Median RT Dose (Range) (Gy)**		
**Focal (Tumor only)**	30.0 (30.0–30.0)	30.0 (30.0–30.0)
**WVI+ PB**	24.0 (23.4–36.0) + 6.0 (0–19.8)	24.0 (23.3–36.0) + 6.0 (0–26.0)
**WB+ PB**	10.0 + 20.0	18.0 + 18.0
**CSI+ PB**	21.6 (19.8–30.6) + 9.4 (5.4–16.2)	23.4 (19.8–24.0) + 16.2 (12.6–27.0)
**Chemotherapy**		
**Yes**	5 (17.2%)	10 (14.9%)
**No**	24 (82.8%)	57 (85.1%)
**Recurrence**		
**Yes**	7 (24.1%)	7 (10.4%)
**No**	22 (75.9%)	60 (89.6%)
**Last reported status**		
**Dead**	2 (6.9%)	0 (0%)
**Alive**	27 (93.1%)	67 (100.0%)

RT, radiotherapy; WVI, whole ventricular irradiation; PB, primary boost; WB, whole brain; CSI, craniospinal irradiation

Although WVI and CSI were the major treatment currently, there was a wide variety of RT field arrangement during the time when patients in our study received treatment. Among the 29 adult patients with IG who received RT, 20 (69.0%) received WVI with PB, six (20.7%) received CSI with PB, two (6.9%) received focal tumor RT only, and one (3.4%) received whole-brain RT (WBRT). Craniotomy and stereotactic biopsy with pathological examination were performed in five (17.2%) and 19 (65.5%) patients, respectively. Five patients (17.2%) did not undergo surgical intervention, but their tumor response was monitored weekly through CT. Five patients (17.2%) received CHT, which included six to ten cycles of etoposide plus carboplatin.

For the 67 pediatric patients with IG (Table 1), the median age at diagnosis was 12.6 years (range, 4.0–17.0 years). The median follow-up time was 7.4 years. There were 17 women (25.4%) and 50 men (74.6%). Two patients (3.0%) had spinal seeding, and 10 patients (14.9%) had ventricular seeding at the time of diagnosis. All 67 pediatric patients underwent RT, of whom 53 (79.1%) received WVI with PB, three (4.5%) received CSI with PB, eight (11.9%) received only focal tumor RT, and three (4.5%) received WBRT. Ten patients (14.9%) received CHT. During the follow-up period, seven patients (10.4%) experienced recurrence, and all of them underwent successful salvage treatment with CSI and CHT. None of the pediatric patients with IG died during the follow-up period.

### Response to therapy and failure patterns in adult patients with IG

**Fig 2:**
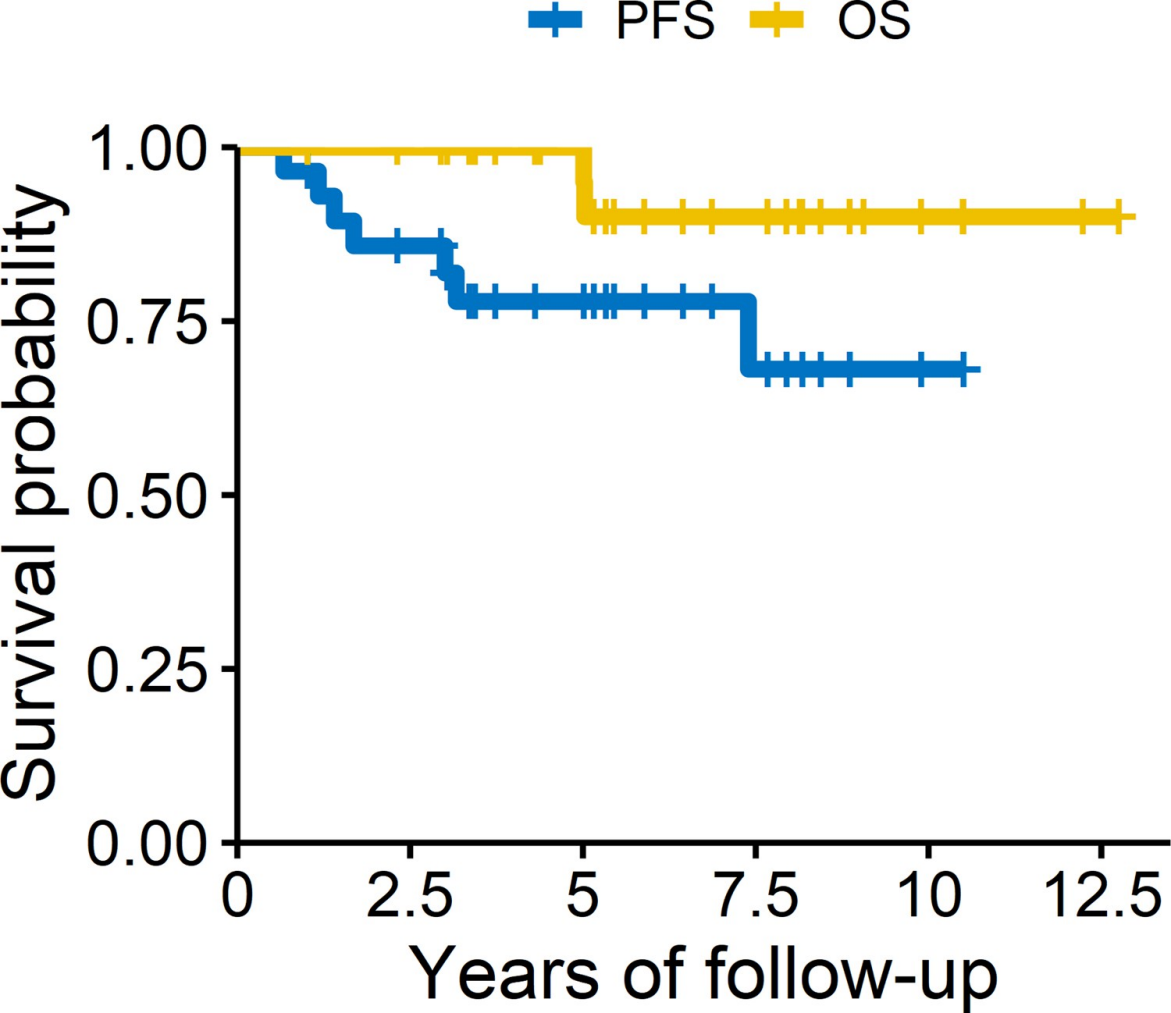
Progression-free survival and overall survival of the cohort.

The PFS and OS of the adult patients with IG after treatment were analyzed and are shown in Fig 2. The 1-, 3-, and 5-year PFS rates were 96.6%, 85.8%, and 77.8%, respectively. The 1-, 3-, and 5-year OS rates were all 100%. Twenty-two patients (75.9%) were successfully treated and had no recurrence. Seven patients (24.1%) experienced recurrence during the follow-up period, detailed information and failure patterns being displayed in Table 2. Among the patients with recurrences, two had received only focal RT, four had received WVI with PB, and one had received WBRT with PB. In terms of tumor recurrence site, two patients experienced recurrence in the ventricular area, both having received only focal RT. Six patients suffered from spinal failure, of whom, at the time of the diagnosis, four had bifocal lesions, one had pineal lesion, and one had suprasellar lesion.

**Table 2:** The detailed information and failure pattern of 7 recurrent patients.

No.	Age at Dx (y/o)	Tumor Location	Initial Seeding	RT	CHT	Recur time (yrs)	Failure area	Salvage Treatment	Status
**1**	22	Bifocal	No	WVI 24 Gy	No	1.2	Spine (T12)	CSI 23.4 Gy + CHT C6	Alive
**2**	26	Bifocal	No	Focal 30 Gy	No	3.0	Left ventricle	CSI 23.4 Gy	Alive
**3**	52	Pineal	No	WVI 24 Gy with PB to 30 Gy	No	1.7	Spine (C2-3, L2, L2-3)	CSI 23.4 Gy	Alive
**4**	19	Bifocal	No	WB 10 Gy with WVI 30 Gy	No	1.4	Spine (L5-S1)	CSI 19.8 Gy + CHT C10	Alive
**5**	20	Supra-sellar	No	Focal 30 Gy	No	7.4	Fourth ventricle and spine (C5-T1)	CSI 23.4 Gy + CHT C6	Alive
**6**	26	Bifocal	No	WVI 24 Gy with PB to 36 Gy	No	0.7	Spine (C3-T1)	CSI 18 Gy + CHT C6	Alive
**7**	38	Bifocal	Yes (Ventricle)	WVI 25.2 Gy with PB to 45 Gy ^a^	Yes	3.2	Spine (Diffuse)	CSI 23.4 Gy + CHT C6	Died

Dx, diagnosis; y/o, years old; RT, radiotherapy; CHT, chemotherapy; Recur time (yrs), Recurrent time at years of follow-up; WVI, whole ventricular irradiation; PB, primary boost; WB, whole brain; CSI, craniospinal irradiation; C6, 6 courses

^a^ Number 7 patient experienced RT interruption for 1 month due to hepatitis flare up.

Among the adult patients with IG who had recurrences, six (85.7%) were successfully treated with salvage RT and CHT. Salvage RT included CSI with a median dose of 23.4 Gy (18–23.4 Gy) and a median PB dose of 29.4 Gy (23.4–36 Gy). Salvage CHT included vinblastine, bleomycin, etoposide, and cisplatin for six to ten courses or etoposide and carboplatin for six to ten courses. Only two patients (6.9%) died after 5 years of follow-up. One patient died after completing salvage therapy due to a hepatitis flare-up, lung and urinary infection, complicated hyponatremia, and seizures. Another patient died without tumor recurrence due to hypercapnic respiratory failure, suspected diabetes insipidus (DI), and central pontine myelinolysis.

### Prognostic factors for adult patients with IG

**Fig 3:**
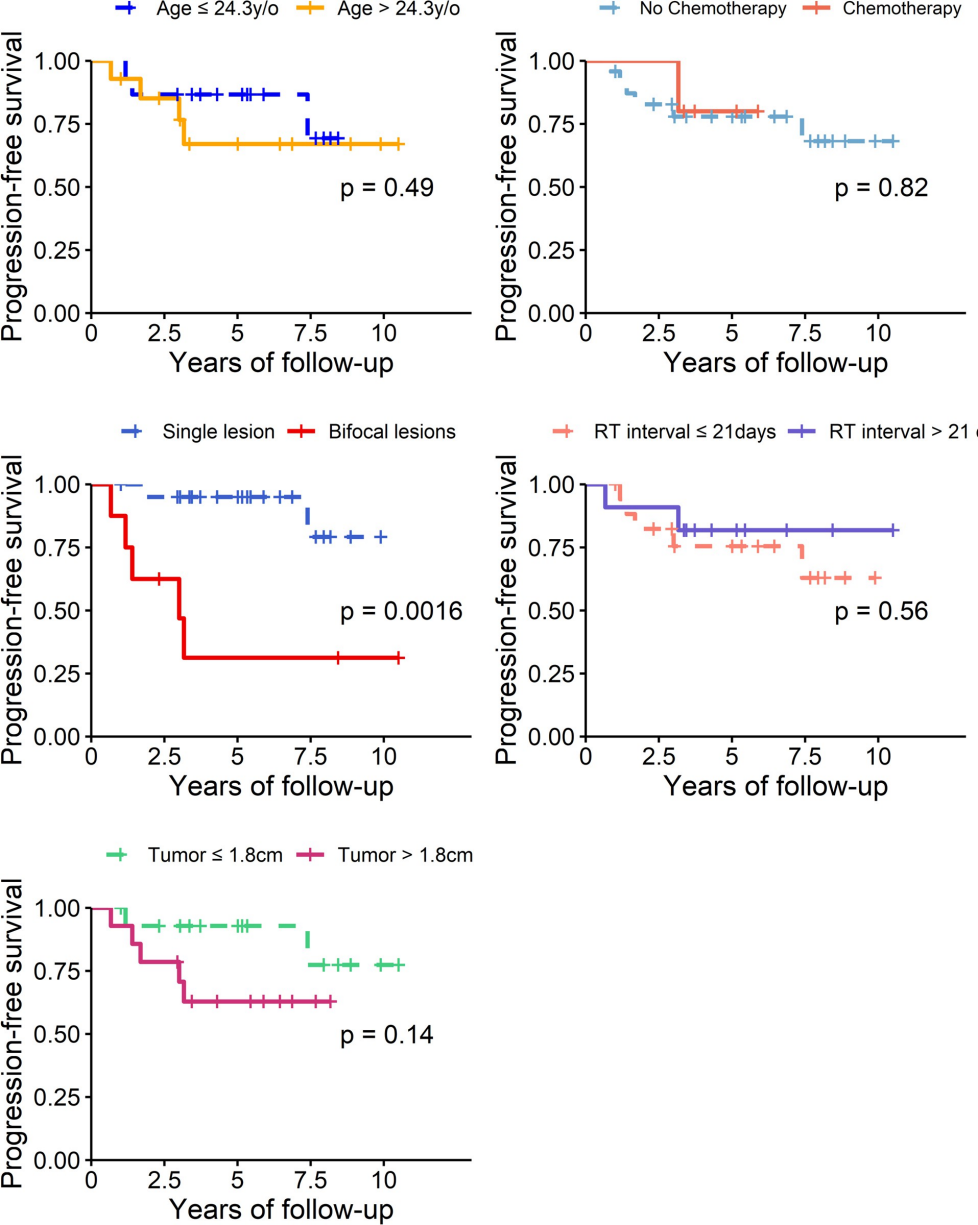
Comparison of progression-free survival curves according to different prognostic factors estimated with the Kaplan-Meier method.

Fig 3 shows the PFS of the adult patients with IG based on different prognostic factors estimated with the Kaplan–Meier method, and the *p*-values were compared using the log-rank test. Patients with bifocal lesions had a significantly lower PFS than those with single lesions (*p =* 0.0016). No significant difference in the PFS was noted when comparing age, tumor size, CHT, or RT interval. Factors including RT field, sex, and seeding status at diagnosis could not be analyzed due to few patient numbers.

Table 3 displays the results of the univariate and multivariate Cox regression analyses of the association between the PFS of adult patients with IG and different variables. In the univariate analysis, bifocal lesions on diagnosis were associated with a significantly lower PFS rate (*p =* 0.008) than single lesions. No significant difference in PFS was noted with respect to age, tumor size, or CHT. Factors including RT field, sex, and seeding status at diagnosis could not be analyzed due to few patient numbers. In the multivariate Cox regression analysis, only bifocal lesions on diagnosis were associated with a significantly lower PFS rate (*p =* 0.008).

**Table 3:** The hazard ratio and *P* value of each factor in single variate and multi-variate Cox model for disease recurrence in adolescent and young adult germinoma patients.

	Univariate analysis		Multi-variate analysis	
Factor	Hazard ratio (95% CI)	*P* value	Hazard ratio (95% CI)	*P* value
**Age**				
> 24.3 years old	1.68 (0.40–7.12)	0.48	0.94 (0.17–5.21)	0.95
≤ 24.3 years old	Reference			
**Tumor size**				
> 1.9 cm	3.26 (0.71–15.09)	0.13	5.76 (0.84–39.69)	0.08
≤ 1.9 cm	Reference			
**Bifocal or single lesions at diagnosis**				
Bifocal lesions	9.09 (1.79–46.33)	0.008	13.58 (2.00–92.29)	0.008
Focal lesion	Reference			
**Chemotherapy**				
Yes	0.78 (0.12–5.10)	0.79	0.41 (0.04–4.38)	0.46
No	Reference			

RT, radiotherapy; CSI, craniospinal irradiation; WB, whole brain; WVI, whole ventricular irradiation

### Post-treatment toxicities in adult patients with IG

There was no secondary malignancy detected during the long-term follow-up. One patient (2.9%) developed DI, one patient (2.9%) reported blurred vision, and one patient (2.9%) reported poor short-time memory after treatment. One patient (2.9%) died of DI-related central pontine myelinolysis without tumor recurrence. This patient was diagnosed with IG without metastasis at the age of 26 years, received WVI with 30 Gy and was followed-up. However, consciousness disturbance, shock with hyponatremia, hypercapnic respiratory failure, and neutropenia were noted in one day. Brain MRI revealed no tumor recurrence hence DI and central pontine myelinolysis were suspected. The patient had a poor response to the treatment and died.

### Comparison between the adult and pediatric patients with IG

**Fig 4:**
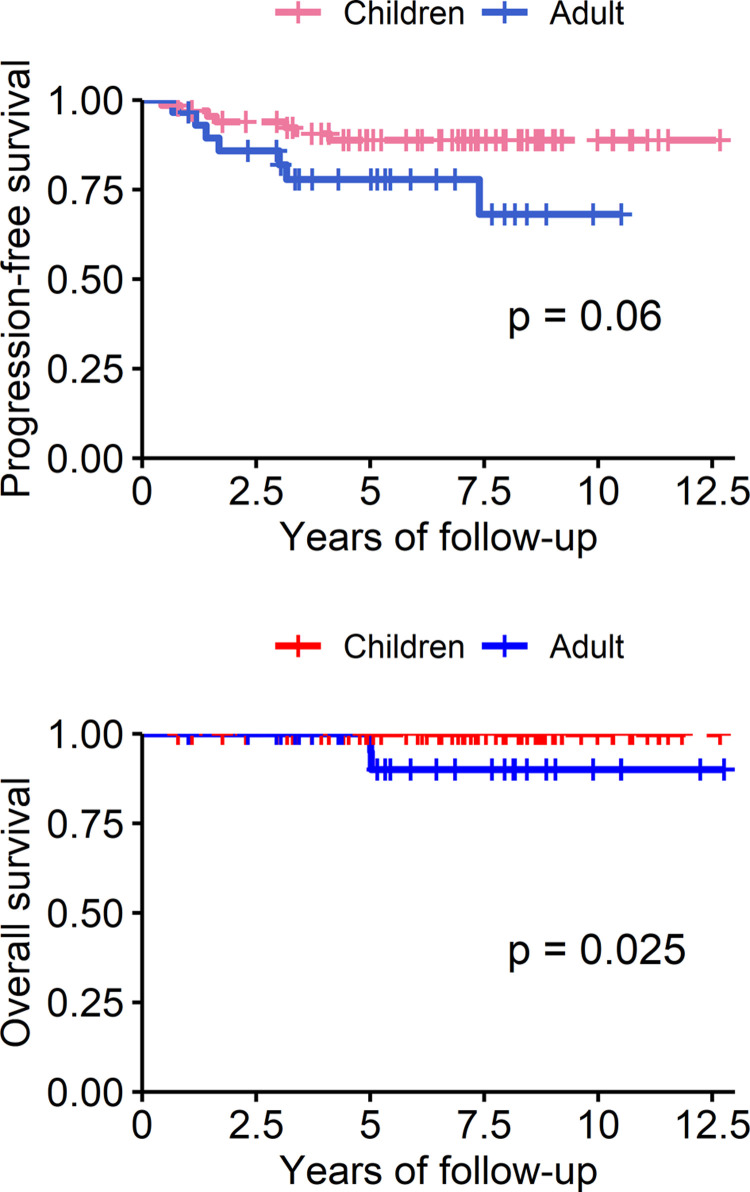
Progression-free survival and overall survival of adult and pediatric patients with intracranial germinoma.

Using the Kaplan–Meier method and log-rank test, we compared the PFS and OS between adult (n = 29) and pediatric (n = 67) patients with IG (Fig 4). Adult patients with IG tended to have a lower PFS (*p* = 0.06) and a significantly lower OS (*p* = 0.025) than pediatric patients. We also compared the PFS and OS in different tumor locations between adult and pediatric IG patients, and the results were presented in S1 Fig.

## Discussion

This study analyzed the treatment outcomes of 29 adult patients with IG. Univariate and multivariate Cox analysis revealed that there was a significant difference in PFS between patients with bifocal and single lesions on diagnosis. Furthermore, adult patients with IG tended to have a lower PFS and a significantly lower OS as compared to the pediatric patients with IG treated in the same period.

Despite the studies on adult patients with IG being limited, inferior treatment outcomes among adolescents and young adults have been reported by several studies [2, 12]. One report claimed that inferior treatment outcomes may be due to decreased patient compliance, less clinical evidence and standardized treatment protocols, and lower clinical trial enrollment rates [2]. Another study using the National Cancer Database in the United States revealed that age was a significant factor for poor treatment outcomes among adolescent and young adult patients with IG [13]. Our study also showed a tendency for inferior PFS and OS among adult patients with IG. Although the mechanism is not clear, one explanation is that the nature of adult and pediatric IG is different. Adult IG may have more metastatic characteristics than pediatric IG. Another possible reason is that because IG is a rare disease especially in adult patients, there are less treatment experience and optimal treatment suggestions for adult IG patients, which may also be one of the reasons for inferior treatment outcomes.

IG originates from progenitor germ cells that erroneously migrate and become trapped in the midline structures of the central nervous system [15]. The two most frequent sites of IGs are the pineal region (51%) and suprasellar region (30%) [12]. Bifocal germinomas, in which IG lesions affect both the pineal gland and suprasellar region, account for 6–26% of patients with IG [16]. It was debated whether patients with bifocal lesions should be treated as having synchronous or metastatic disease. For instance, in America it is considered a metastatic disease, while in Europe it is seen as a localized disease [17]. Studies have shown that bifocal germinoma is a synchronous disease due to a good tumor response and a similar control rate after limited irradiation. Huang et al. reported the cases of seven pediatric patients with bifocal IG who received WVI without CHT. The 2- and 5-year survival rates were all 100% [18], and there was no significant difference in OS or DFS between patients with bifocal or single lesions [18]. Lafay-Cousin et al. considered bifocal IG a local-regional disease, and suggested that CHT with focal RT was sufficient to provide good tumor control [19]. Cuccia et al. analyzed five cases of suprasellar/pineal bifocal germ cell tumors and stated that the presence of two tumors does not indicate dissemination [20].

Conversely, some studies have reported that bifocal IG behaves like a metastatic disease since spinal cord failure were seen in some patients who received limited field RT [6, 21–23]. Ogawa et al. reported the cases of 18 patients with bifocal IG, three of whom had spinal recurrences (11.1%) [23]. Phi et al. claimed that bifocal IGCTs may result from the metastatic spread of suprasellar or pineal IGCTs, and tumor seeding was significantly associated with bifocal lesions [21]. Weksberg et al. collected information on 55 cases of bifocal IG from literature and their institute and found that three of them suffered spinal cord failure after receiving limited field RT with or without CHT. In contrast, there were no spinal cord failures in patients receiving CSI [22]. Lian et al. reported 23 patients with bifocal IG treated with CSI, none of whom experienced spinal cord failure, while four (16.7%) of 24 patients treated with WVI or WBRT developed spinal seeding [6]. In our study, seven adult patients with IG (24.1%) suffered from recurrence; at the time of the diagnosis, five of them had bifocal disease without dissemination and received WVI without CHT, and all of them suffered from spinal failure, indicating the metastatic potential of this disease. We think bifocal lesions among adult IG patients include synchronous as well as metastatic characteristics, and bifocal lesions with only synchronous characteristic could be controlled by limited field RT. However, lesions with metastatic characteristic may result in failures over areas which could not be adequately covered by limited field RT, such as spinal cord. Therefore, in order to prevent spinal recurrences, we recommend CSI or WVI with CHT for adult patients with bifocal IG. However, if adult patients with IG with bifocal lesions receive only WVI, frequent spine MRI follow-up is recommended.

For pediatric patients, performing CSI with a high irradiation dosage result in neural toxicities [24, 25], such as impaired neurocognitive development, impaired intellectual functions, or poor pituitary function [18, 25]. A previous study also reported a significant correlation between receiving RT at a younger age and late cognitive impairments [26]. For adult patients, the neurological development is already complete, so the damage to neurocognitive functions after RT is less severe than that in pediatric patients, and the toxicity is generally tolerable [27]. Foote et al. reported that low-dose CSI with a field boost is effective with minimal morbidity in adult patients with IG [9]. Sutton et al. also reported that the quality-of-life assessments of 22 adult IG survivors who received low-dose CSI were generally good [28]. Since neurotoxicity is less harmful to adult patients with IG, CSI could be considered as a tolerable option for adults with bifocal IG.

The treatment benefits of using CHT in adult patients with IGs remain unknown. Some institutes showed excellent OS with CHT and WVI, even without tumor boost [29]. One study from Korea reported that upfront CHT was used before response-adapted RT; the treatment result was good, but CHT-induced side effects, such as grade 3 neutropenia (97.8%), anemia (40.7%), and thrombocytopenia (67.1%), were also reported [30]. Our study did not show a significant association between CHT and PFS in adult patients with IG. Further studies are needed to evaluate the benefits of CHT.

Our study has several limitations. First, there may be bias because of the retrospective nature of study. Second, our study lacked pre-treatment and post-treatment neurocognitive evaluations of the adult patients. Third, the number of patients was limited. However, due to the scarcity of adult cases of IG, it is difficult to identify and include more patients or perform a clinical trial.

In conclusion, our study showed that bifocal lesions on diagnosis were associated with lower a PFS than single lesions, and adult patients with IG tended to have a poorer PFS and OS compared to pediatric patients with IG. Since information on prognosis and optimal treatment for adult patients with IG is scarce, our findings can help oncologists plan optimum treatment strategies for them.

## Supporting information

S1 Fig Progression-free survival and overall survival of different tumor locations between adult and pediatric patients with intracranial germinoma. Although there is no significantly difference, adult intracranial germinoma (IG) patients with bifocal lesions and suprasellar lesion tended to have poorer progression-free survival and overall survival compared to pediatric IG patients. However, there are only 4 patients with bifocal lesions in pediatric IG patients, and only 2 patients with basal ganglia lesions in adult IG patients. Because of few patient numbers of these two subgroups, the interpretation of may be just as a reference.
(PDF)
