# Supplementary material for: Bifocal lesions have a poorer treatment outcome than a single lesion in adult patients with intracranial germinoma
Source: PLoS One. 2022 Mar 1;17(3):e0264641. doi: 10.1371/journal.pone.0264641 (PMC8887760; doi:10.1371/journal.pone.0264641)
Supplement: S1 Fig — Although there is no significantly difference, adult intracranial germinoma (IG) patients with bifocal lesions and suprasellar lesion tended to have poorer progression-free survival and overall survival compared to pediatric IG patients. However, there are only 4 patients with bifocal lesions in pediatric IG patients, and only 2 patients with basal ganglia lesions in adult IG patients. Because of few patient numbers of these two subgroups, the interpretation of may be just as a reference. (PDF) [file pone.0264641.s001.pdf]

Supporting information

S1 Fig.

Progression-free survival and overall survival of different tumor locations between adult and pediatric patients with intracranial germinoma.

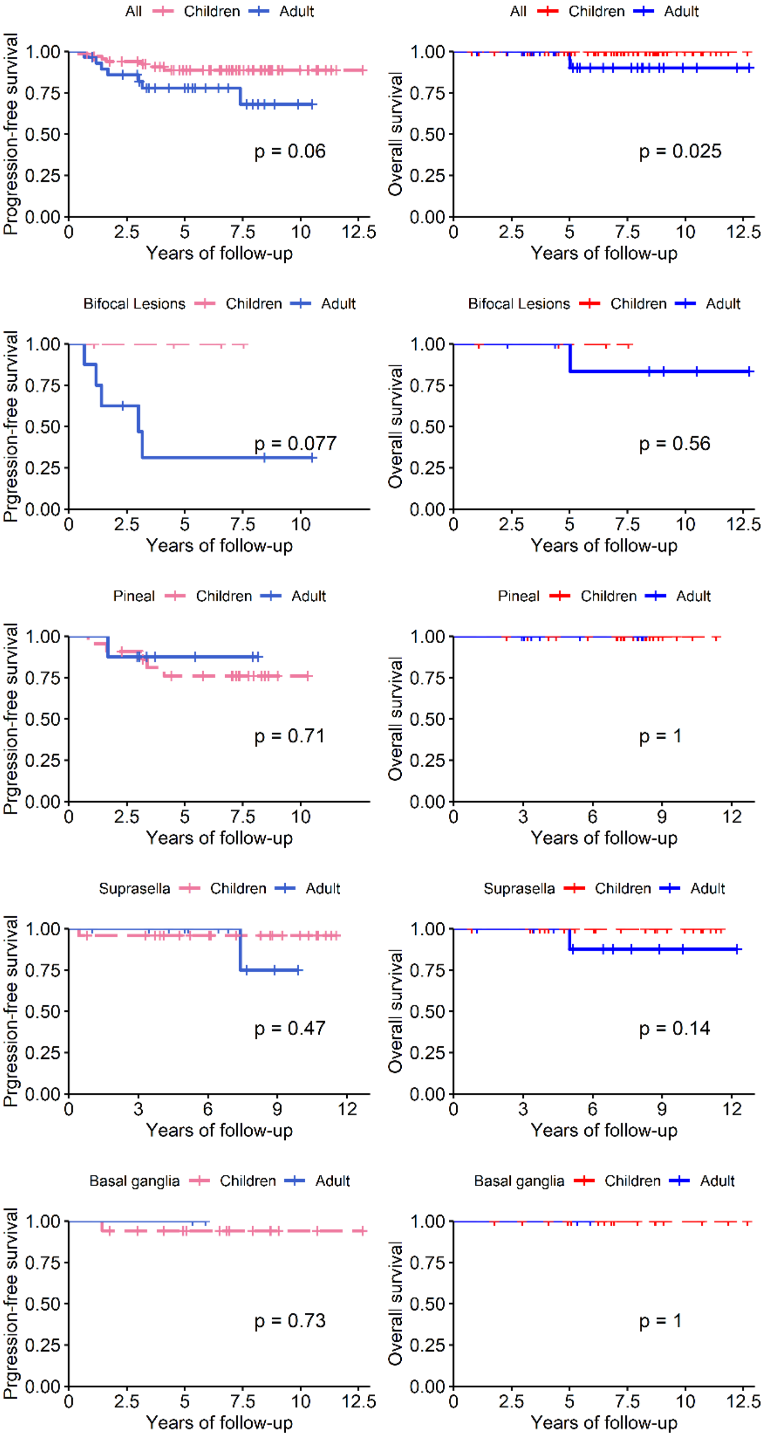

Although there is no significantly difference, adult intracranial germinoma (IG) patients with bifocal lesions and suprasellar lesion tended to have poorer progression-free survival and overall survival compared to pediatric IG patients. However, there are only 4 patients with bifocal lesions in pediatric IG patients, and only 2 patients with basal ganglia lesions in adult IG patients. Because of few patient numbers of these two subgroups, the interpretation of may be just as a reference.
